# Long-term effectiveness of non-surgical open-bite treatment: a systematic review and meta-analysis

**DOI:** 10.1186/s40510-023-00467-2

**Published:** 2023-06-01

**Authors:** Maria-Zoi Theodoridou, Athanasia-Eirini Zarkadi, Vasileios F. Zymperdikas, Moschos A. Papadopoulos

**Affiliations:** 1 Department of Public Health, Faculty of Medicine, European University of Cyprus, Nicosia, Cyprus; 2 Department of Paediatric Dentistry, School of Dentistry, Scholl of Health Sciences, Aristotle University of Thessaloniki, Thessaloniki, Greece; 3 Department of Orthodontics, Faculty of Dentistry, School of Health Sciences, Aristotle University of Thessaloniki, Thessaloniki, Greece; 4 Dental Department, 424 Military Hospital of Thessaloniki, Thessaloniki, Greece; 5 Department of Midwifery, School of Health Sciences, University of Western Macedonia, Ptolemaida, Greece

## Abstract

**Background:**

The etiology of open bite is complex, involving various genetic or environmental factors. Several treatment alternatives have been suggested for the correction of open bite, yet their long-term effectiveness remains controversial.

**Objective:**

To assess the long-term effectiveness of open-bite treatment in treated with non-surgical approaches versus untreated patients, through lateral cephalometric radiographs.

**Search methods:**

Unrestricted search of 16 electronic databases and manual searches up to November 2022.

**Selection criteria:**

Randomized or non-randomized controlled trials reporting on the long-term effects of open-bite treatment through angular lateral cephalometric variables.

**Data collection and analysis:**

Only angular variables on lateral cephalometric radiographs were considered as primary outcomes. For each outcome, the mean differences and 95% confidence intervals were calculated using the random-effects model to consider existing heterogeneity. The revised Cochrane risk-of-bias tool (R.o.B. 2.0) and the risk-of-bias tool for non-randomized studies for interventions (ROBINS-I) were utilized for the randomized and non-randomized trials, respectively.

**Results:**

From the initially identified 26,527 hits, only 6 studies (1 randomized and 5 retrospective controlled trials) were finally included in this systematic review reporting on 244 open-bite individuals (134 patients and 110 untreated controls), while five of them were included in the meta-analyses, assessing either the interval ranging from treatment start to post-retention (T3–T1) or from end of treatment to post-retention period (T3–T2). Regarding the vertical plane, for the T3–T2 interval, no significant differences were found for the assessed skeletal measurements, indicating a relative stability of the treatment results. Similarly, with regard to the T3–T1 interval, no significant differences could be identified for the examined skeletal variables, implying that the produced effects are rather minimal and that the correction of the open bite was performed mainly through dentoalveolar rather than skeletal changes. Further, no significant changes could be identified regarding the inclination of the upper and lower incisors. Only the nasolabial angle was significantly reduced in the treated patients in the long term.

**Conclusions:**

According to existing evidence, the influence of non-surgical treatment of open bite on the skeletal tissues and the inclination of the incisors is rather minimal in the long term, while only the nasolabial angle was significantly reduced.

**Supplementary Information:**

The online version contains supplementary material available at 10.1186/s40510-023-00467-2.

## Introduction

### Rationale

Open bite is considered as a deviation in the vertical relationship of the maxillary and mandibular dental arches, characterized by a lack of contact between opposing segments of teeth [1]. It is reported to be a common type of malocclusion and it can be classified as skeletal or dentoalveolar, based on the affected dental tissues or as anterior, posterior or lateral according to the dental arch section that the malocclusion is developed. The etiology of open bite is complex, involving genetic factors or diseases, environmental factors as well as oral habits [2–5].

Several alternatives have been proposed for the orthodontic non-surgical treatment of open bite, including intrusion or vertical control of posterior teeth [6–8], or extrusion of the anterior teeth [9]. However, in unfavorable skeletal patterns, an orthognathic surgery is suggested as the most appropriate approach [10, 11]. A variety of fixed or removable appliances have been implemented for the orthodontic management of open bite [12–14], yet their effectiveness regarding long-time treatment outcomes is rather controversial [2, 15–18].

In detail, while some trials report on significant relapse following non-surgical open-bite treatment via various fixed or removable appliances [8, 9, 18] there are also reports of relatively stable results after molar intrusion through skeletal anchorage [14], or treatment with four premolar extractions [19]. However, the absence or the inclusion of inappropriate control groups (i.e., matched untreated open-bite patients) in all previously described studies does not permit safe conclusions for the described treatment outcomes. Similar to the latter, the relative published systematic reviews summarize results from both controlled and uncontrolled trials, which implies a cautious interpretation of the reported results [3, 13, 20, 21].

### Objective

Thus, this study aims to summarize current evidence exclusively from randomized controlled trials (RCTs), prospective controlled clinical trials (pCCTs) and retrospective clinical trials (rCCTs) regarding the long-term stability of non-surgical open-bite treatment compared with untreated matched controls, through lateral cephalometric records, as well as to identify any factors potentially affecting the treatment outcomes.

## Materials and methods

### Protocol and registration

The present review is constructed a priori according to the Cochrane Handbook for Systematic Reviews of Interventions [22], while the results are reported in accordance with the PRISMA statement [23] and the corresponding extension for abstracts [24]. The respective protocol was registered in PROSPERO (Registration Number: CRD42021251576).

### Information sources and search

Sixteen electronic databases, including, among others, PubMed, Scopus, Cochrane Database of Systematic Reviews, Nature, Ovid, Google Scholar, were systematically and unrestrictedly searched up to November 2022. MESH terms and the respective keywords were used properly to fit each database (Additional file 1: Table S1). The search strategy did not include any limitations concerning language, publication year, or status. The exact strategy for each database was conducted by the first two review authors (MZT and AEZ), with the guidance and supervision of the last author (MAP). The reference lists of the relevant reviews were planned to be manually searched as well, for the identification of potentially eligible trials. The gray literature was also explored through proper registers and databases. When considered necessary, authors were contacted for complementary/missing data or clarifications. The search was performed independently by two authors (MZT and AEZ).

### Eligibility criteria and study selection

The eligibility criteria were pre-determined, and they were defined according to the PICOS approach, (Problem/Patients/Population, Intervention/Indicator, Comparison, Outcome, and Study Type/Design) (Table 1) [22, 25, 26]. A study was considered eligible when it reported on open-bite patients (Patients) treated with non-surgical alternatives (Interventions) and compared with untreated control samples of matched individuals (Comparison). These studies had to be randomized or non-randomized controlled trials (Study Design) as well as to report on angular cephalometric variables from at least one year after the end of the treatment (Outcome). Linear cephalometric variables (Outcome) would be included in the analyses only if the magnification factor of the respective radiographic machines were reported in the original studies. In order for a trial to be considered eligible simultaneously all of the inclusion and none of the exclusion criteria had to be fulfilled. Studies reporting on results immediately after the end of the treatment or short-term post-treatment outcomes were not included. After the elimination of duplicates, all remaining articles were sequentially screened on the basis of title, abstract, and full text. For trials published in several languages, the English version was assessed.

**Table 1:** Eligibility criteria used for the selection of the studies according to the PICOS approach

Category	Inclusion criteria	Exclusion criteria
Participant characteristics	Studies on human patients with open-bite malocclusion of any age or gender	Patients with craniofacial syndromes and/or cleft lip palate
		Patients with temporomandibular joint disorders
		Animal studies
		Patients with deep bite
Intervention	Orthodontic treatment with fixed or removable appliances	Surgical treatment of open bite
Comparison	At least one control group with no treated open-bite patients	No untreated control group
		Patients treated for open bite with different treatment alternatives
Outcome	Studies providing angular skeletal, dentoalveolar and soft tissue cephalometric measurements from lateral cephalometric analysis at least one year after the end of the treatment	Studies providing linear measurements on lateral cephalograms without reporting the respective magnification factor
	Studies providing linear measurements on lateral cephalograms and reporting the respective magnification factor	Electromyographic evaluation
		Cost benefit analyses
		Studies without long-term results on open bite
		Ongoing studies
Study design	Randomized controlled clinical trials	Unsupported opinion of expert
	Prospective controlled clinical trials	Editor’s choices
	Retrospective controlled clinical trials	Replies to the author/editor
		Interviews
		Commentaries
		Books’/conferences’ abstracts
		Summaries
		In vitro/in silico
		Studies with missing or inappropriate data
		Studies with no English abstract
		Cross-sectional surveys
		Case series without control or with inappropriate control group
		Case reports or reports of cases
		Case control observational studies
		Cohort studies
		Narrative reviews^a^
		Systematic reviews^a^
		Meta-analyses^a^

^a^After checking the reference lists for relevant article

### Data collection process and data items

Data were extracted independently by two authors (MZT and AEZ) on predefined and piloted forms prepared by the third author (VFZ). Any ambiguities were resolved after discussion with the last author (MAP). In order to assess the stability of open-bite treatment, only angular variables on lateral cephalometric radiographs were considered as primary outcomes since they are not affected by different magnification factors. In contrast, linear measurements were not included in the analysis since they are prone to magnification bias, unless the magnification factor of the respective radiographic equipment was provided [27]. Further, the extracted data were planned to be classified according to time evaluation of effects in two difference groups: (a) T3–T1 period ranging from the beginning of the treatment until the post-retention interval, or (b) T3–T2 period between the end of the treatment and the post-retention interval. Since a considerable variability was presumed among the different similar terms used among the included trials for the assessed measurements, all equivalent terms of a specific variable were grouped into one (Additional file 1: Table S2) and only this single term was used throughout the review.

### Risk of bias in individual studies

The risk of bias for the individual studies was assessed separately for randomized and non-randomized trials.

For the *randomized trials*, the revised Cochrane risk-of-bias tool (R.o.B. 2.0) [28] was implemented, which was composed of five domains: 1. bias arising from the randomization process, 2. bias due to deviations from intended interventions, 3. bias due to missing outcome data, 4. bias in the measurement of the outcome, and 5. bias in the selection of the reported result. Each domain was rated with one of the following ratings: «low risk», «some concerns» or «high risk». Finally, the overall risk of bias for the respective study was considered as: «low risk» (when all domains were found to present «low risk»), «some concerns» (when at least one domain was judged with «some concerns») or «high risk» (when at least one domain was found to present «high risk» or when multiple domains were judged with «some concerns»).

For the *non-randomized trials*, the risk-of-bias tool for non-randomized studies for interventions (ROBINS-I) was used [29]. The latter was comprised of seven domains: 1. bias due to confounding, 2. bias in the selection of participants into the study, 3. bias in classification of interventions, 4. bias due to deviations from intended interventions, 5. bias due to missing data, 6. bias in the measurement of outcomes, and 7. bias in the selection of the reported result. Each of the aforementioned domains was given one of the following ratings: «low risk», «moderate risk», «serious risk», «critical risk» or «no information». A rating regarding the overall risk of bias for the respective study was reached as follows: «low risk» (when all domains were rated with «low risk»), «moderate risk» (when all domains were rated with «low risk» and at least one with «moderate risk»), «serious risk» (when at least one domain was rated with «serious risk», but no domain was rated with «critical risk»), «critical risk» (when at least one domain was rated with «critical risk»), and «no information» (when no domain was rated with «serious risk» or «critical risk» and at least one domain was rated with «no information»). Risk-of-bias assessment was performed independently by two review authors (MZT and AEZ).

### Risk of bias across studies

If an adequate number of trials were found (at least 10), reporting biases (publication bias and/or “small study effects”) were planned to be assessed through the visual inspection of contour-enhanced funnel plots [30], Begg’s rank correlation test [31] and Egger’s weighted regression test [32]. The Duval and Tweedie trim and fill procedure [33] was planned to be performed in case the latter hinted towards the existence of publication bias. Moreover, for each included trial, the articles were carefully examined in order to identify potential missing outcomes and/or outcomes that were originally declared to be assessed but were eventually not reported. In this respect, several registers were also explored for the possible existence of the original protocols of the respective trials in order to identify any differences between the methods and outcomes reported on the protocols and the ones presented in the published studies.

## Summary measures and synthesis of results

Data were judged as suitable for pooling if similar control groups of untreated patients with open bite were found, providing data on the same angular measurements on lateral cephalometric radiographs. Mean differences and the corresponding 95% confidence intervals (CIs) were calculated for each outcome. A random-effects model as the one proposed by DerSimonian and Laird [34] was implemented, because it takes into account existing heterogeneity and in the present review the samples were suspected to be heterogeneous due to potential variations of the patient characteristics (age, sex) as well as the exact treatment protocols followed (type of appliance, post-treatment interval duration). For variables assessed in less than five studies, exploratory analyses were undertaken. Moreover, the extent and impact of between-study heterogeneity were assessed through the inspection of forest plots and by calculating the *τ*^2^ and *I*^2^ statistic, respectively. Based on the *I*^2^ scores, heterogeneity was considered as probably not important (0–40%), moderate (30–60%), substantial (50–90%) or considerable (70–100).

### Additional analyses

Mixed effects subgroup analyses were planned in order to explore possible sources of heterogeneity, based on a priori determined factors. These factors were classified as patient-(gender, skeletal maturity stage, dentition) or treatment-related (exact type of appliance used, treatment duration). In an effort to minimize the risk of excessive significance testing, these analyses would be performed when the respective factor was reported in at least five included trials.

The overall quality of evidence for each of the primary outcomes was planned to be rated using the Grades of Recommendation, Assessment, Development, and Evaluation (GRADE) approach [35] according to the following interpretations: “high quality”: we are very confident that the true effect lies close to that of the estimate of the effect, “moderate quality”: we are moderately confident in the effect estimate: the true effect is likely to be close to the estimate of the effect, but there is a possibility that it is substantially different, “low quality”: our confidence in the effect estimate is limited: the true effect may be substantially different from the estimate of the effect, and “very low quality”: we have very little confidence in the effect estimate: the true effect is likely to be substantially different from the estimate of effect.

Furthermore, for meta-analyses involving at least ten trials, the robustness of the respective results was assessed via sensitivity analyses according to: 1. the study design and 2. the quality rating estimate of the GRADE analysis.

All analyses were performed in RStudio Version 3.3.3 (RStudio, Inc., Boston, USA) using the “meta” package. All P values were two-sided with *α* = 5%, with the exception of the test of between-studies heterogeneity with *a* = 10% [36].

## Results

### Study selection

**Fig. 1:**
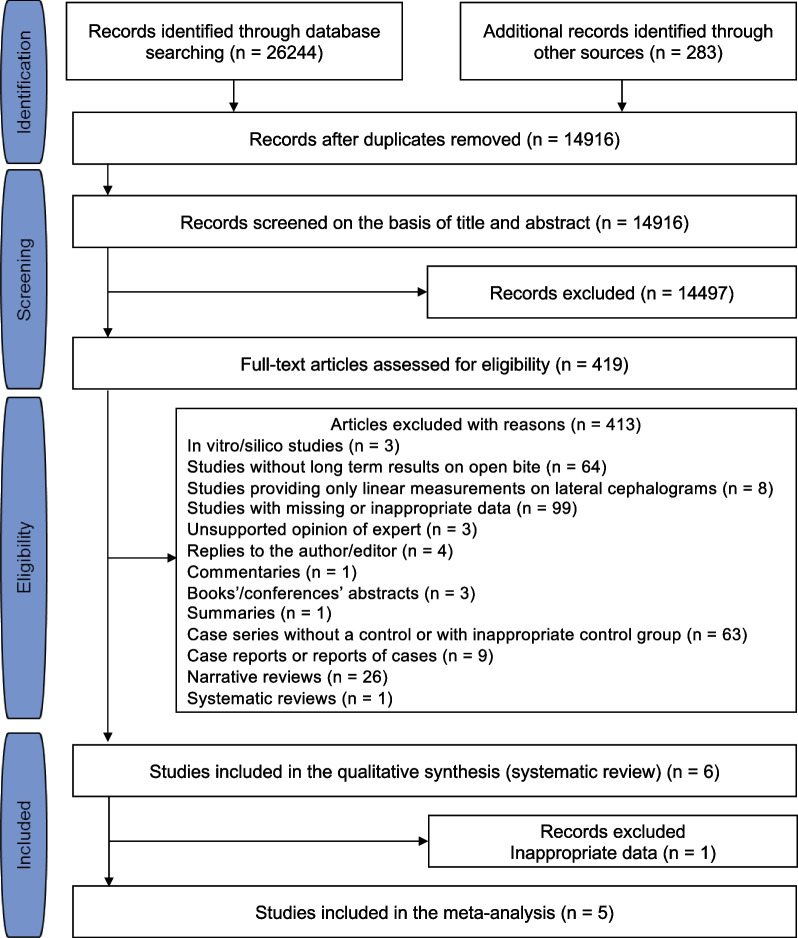
PRISMA flow diagram depicting the process followed for the selection of the studies

Among the initially retrieved 26,527 records, following the removal of duplicates and sequential elimination on the basis of title, abstract and full text (Additional file 1: Table S3), six studies [37–42] were finally included in the systematic review for a qualitative evaluation, while only five of them could be included in the meta-analyses for a quantitative evaluation (Fig. 1), due to missing outcomes in the study of Fränkel and Fränkel [40] that could not be retrieved. In total, 17 authors were contacted, for missing data and/or additional clarifications, while only two of them replied back and provided the requested information (details available upon request).

### Study characteristics and risk of bias within studies

The characteristics of the six included studies are summarized in brief in Table 2. All of them took place at a university setting, reporting on 244 open-bite subjects, including 134 patients that received treatment for the correction of open bite, and 110 untreated open-bite matched controls. Data regarding the age and/or the follow-up time were not reported in all eligible trials, while the pertinent treatment modalities for open-bite management were different in each study, including various pertinent fixed and removable appliances. All included studies provided data regarding both skeletal and dentoalveolar changes, while three articles provided additional soft tissue cephalometric outcomes. None of the studies provided information regarding the magnification factor of the implemented radiographic equipment, thus only angular cephalometric variables were considered eligible.

**Table 2:** Characteristics of the 6 studies included in the current systematic review

	Article	Study Design	Setting	Characteristics of patients	Interventions	No of patients (M/F)	Age in years (SD)	Tx time (SD)^a^ (y)	Follow-up time (SD) (y)	Outcomes	Conclusions	Risk of bias
1	Cozza et al. [37]	rCCT	University; Italy	Presence of thumb-sucking habits before treatment, constricted maxillary arch, negative overbite, fully erupted permanent first molars and permanent incisors, no permanent teeth extracted prior to or during treatment, two consecutive lateral cephalograms of good quality obtained before treatment and again approximately 2 years after the completion of treatment, pre-treatment value for mandibular plane angle (MPA) relative to Frankfort horizontal of 25° or greater	Quad-helix/Crib	Exp: 21 (6/15) Ctr: 21 (10/11)	Exp: 8.4 (1.5) Ctr: 8.6 (0.9)	Exp: 1.5 (0.6) Ctr: total observation time was 3.0 (1.1) years	Exp: 2.0 (NR) Ctr: total observation time was 3.0 (1.1) years	Skeletal Dental Soft tissue	The appliance was effective in correcting dental open bite in 85% of the growing subjects with thumb-sucking habits and dentoskeletal open bites, with a clinically significant improvement in maxillomandibular vertical skeletal relationships	Low
2	Defraia et al. [38]	rCCT	University; Italy	Initial mandibular plane angle relative to the Frankfort horizontal (MPA) 25° or greater, 2 consecutive lateral cephalograms of good quality with adequate landmark visualization and minimal or no rotation of the head, taken before treatment and after therapy and retention, no permanent teeth extracted before or during treatment	Open-bite Bionator	Exp: 20 (11/9) Ctr: 23 (10/13)	Exp: 8.3 (0.8) Ctr: 9.1 (1.6)	Exp: 1.5 (NR) Ctr: total observation time was 2.8 (1.1) years	Exp: 1 (NR) Ctr: total observation time was 2.8 (1.1) years	Skeletal Dental Soft tissue	Early treatment e with the open-bite bionator mainly consists of significant improvement in intermaxillary divergence, without favorable effects on the extrusion of posterior teeth	Low
3	Ferreira et al. [39]	RCT	University; Brazil	Previous treatment of Angle’s Class I malocclusion and AOB, for 12 months, upper permanent first molars in occlusion, no dental agenesis or permanent teeth loss, no dental extractions	Removable appliance with palatal crib associated with high-pull chincup therapy	Exp: 19 (6/13) Ctr: 19 (2/17)	Exp: (NR) Ctr: (NR)	Exp: 1 Ctr: 1	1.3 (NR)	Skeletal Dental Soft tissue	The early open-bite treatment with a removable appliance and palatal crib associated with high-pull chincup therapy provided stability of 95%	High
4	Fränkel and Fränkel [40]	rCCT	University; Germany	Hyperdivergent skeletal pattern, large interlabial distance and postural weakness of the orofacial muscles, skeletal development in the craniofacial complex observed in approximately analogous growth periods, from the first stage of the mixed dentition through the pubertal growth spurt	Functional regulators developed by Fränkel	Exp: 30 (NR) Ctr: 11 (NR)	NR	NR	> 4 (NR)	Skeletal Dental	Some dentofacial deformities in the skeletal open-bite cases were corrected to the average norms and a considerable change in the soft tissue profile occurred	Moderate
5	Mucedero et al. [41]	rCCT	University; Italy	No sucking habits, overbite < 0 mm, posterior transverse interarch discrepancy ≥ 3 mm, Frankfort horizontal to mandibular plane angle > 26°, full eruption of first permanent molars and of maxillary and mandibular incisors, no permanent teeth extracted	RME and Posterior bite block and fixed appliances	Exp: 16 (2/14) Ctr: 16 (2/14)	Exp: 8.1 (1.1) Ctr: 8.3 (1.2)	Exp: 1.6 (0.5) Ctr: 1.3 (0.8)	Exp: 3.9 (1.7) Ctr: 3.7 (1.7)	Skeletal Dental	This protocol led to successful and stable positive overbite in 100% of the patients and it was associated with reduced extrusion of maxillary and mandibular molars and a significant improvement in vertical skeletal relationships	Low
6	Mucedero et al. [42]	rCCT	University; Italy	Thumb-sucking habit before treatment, negative overbite, constricted maxillary arch as consequence of thumb sucking, full eruption of first permanent molars and permanent incisors, no permanent teeth extracted, postpubertal skeletal maturity at T3 (CS 4–6)	Quad-Helix/crib appliances and fixed appliances	Exp: 28 (11/17) Ctr: 20 (10/10)	Exp: 8.2 (1.3) Ctr: 8.1 (0.4)	Exp: 1.5 (0.4) Ctr: 1.7 (0.4)	Exp: 4.9 (1.3) Ctr: 4.7 (0.6)	Skeletal Dental	In the long term, the use of this appliance led to successful outcomes in about 93% of the patients and it was associated with significant downward rotation of the palatal plane	Low

*Ctr* control group, *Exp* treatment group, *M/F* males/females, *NR* not reported, *rCCT* retrospective controlled clinical trial, *RCT* randomized clinical trial; controlled trial, *RME* rapid maxillary expansion; *y* years

^a^Control group received no intervention; it refers to the observation time

Regarding the risk-of-bias assessment, the RCT of Ferreira et al. [39] was found to present a high risk of bias (Table 3; Additional file 1: Table S4a), while only one rCCT was judged with a moderate risk of bias [40] and the remaining rCCTs [37, 38, 41, 42] were found to present a low risk of bias (Table 4; Additional file 1: Table S4b).

**Table 3:** Risk-of-bias assessment of the randomized controlled trials according to the revised Cochrane risk-of-bias tool for (RoB 2.0)

Study	Risk of bias arising from the randomization process	Risk of bias due to deviations from the intended interventions (effect of assignment to intervention)	Missing outcome data	Risk of bias in measurement of the outcome	Risk of bias in selection of the reported result	Overall risk of bias
Ferreira et al. [39]	Some concerns	Some concerns	Low risk	Low risk	Some concerns	High risk

**Table 4:** Risk-of-bias assessment of the non-randomized trials according to the ROBINS-I tool

Study	Bias due to confounding	Bias in selection of participants into the study	Bias in classification of interventions	Bias due to deviations from intended interventions	Bias due to missing data	Bias in measurement of outcomes	Bias in selection of the reported result	Overall bias
Cozza et al. [37]	Low risk	Low risk	Low risk	Low risk	Low risk	Low risk	Low risk	Low risk
Defraia et al. [38]	Low risk	Low risk	Low risk	Low risk	Low risk	Low risk	Low risk	Low risk
Fränkel and Fränkel [40]	Moderate risk	Low risk	Low risk	Low risk	Low risk	Low risk	Low risk	Moderate risk
Mucedero et al. [41]	Low risk	Low risk	Low risk	Low risk	Low risk	Low risk	Low risk	Low risk
Mucedero et al. [42]	Low risk	Low risk	Low risk	Low risk	Low risk	Low risk	Low risk	Low risk

### Results of individual studies and synthesis of results

As explained above, the reported effects from the original studies were classified on the basis of the assessed time intervals in two different groups. In the current investigation, meta-analyses were performed in order to assess the long-term effectiveness of open-bite treatment compared to untreated patients with open bite, for both assessed treatment periods. However, since each of these meta-analyses included data from a maximum of four original studies, they should be probably regarded as exploratory analyses. The variables that were included only in the qualitative analysis of the current investigation, are reported in Tables 5 and 6.

**Table 5:** Details of the studies included only in the qualitative analysis (systematic review), regarding the angular cephalometric variables for the T3–T2 period

Variables	Ferreira et al. [39]
*Skeletal cephalometric variables*	
SN.PP	NS
SN/GoGn	NS
NS.Gn	NS
*Dentoalveolar cephalometric variables*	
U1.NA	NS
L1.NB	SSE
*Soft tissue cephalometric variables*	
Nasolabial angle	NS
Mentolabial angle	NS
Gl.Sn.P’	NS

*NS* not significant, *SSE* statistically significant in favor of the experimental group, *SSC* statistically significant in favor of the control group

**Table 6:** Details of the studies included only in the qualitative analysis (systematic review), regarding the angular cephalometric variables for the T3–T1 period

Variables	Fränkel and Fränkel [40]
*Skeletal cephalometric variables*	
SN.PP	SSC
MPA	SSE
ArGoMe	SSE
The total of the facial angles (Σ) (°) (Jarabak)	SSE

*NS* not significant, *SSE* statistically significant in favor of the experimental group, *SSC* statistically significant in favor of the control group

#### Assessment of treatment effects for T3–T2 period (post-retention to post-treatment)

**Fig. 2:**
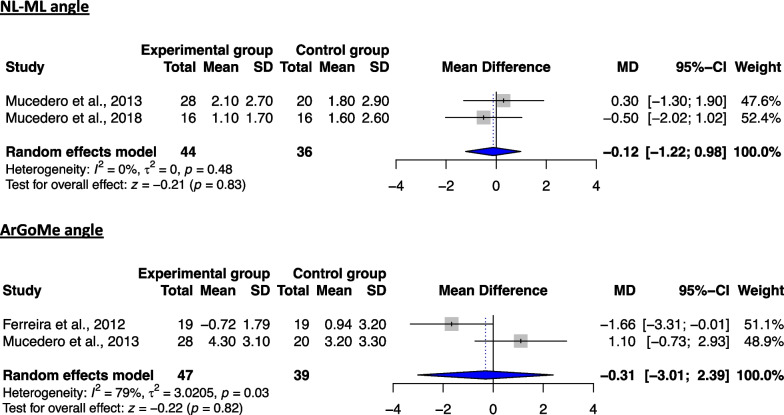
Forest plot of the mean difference of the vertical plane angles (in °) between treated and control groups for the T3–T2 interval

**Fig. 3:**
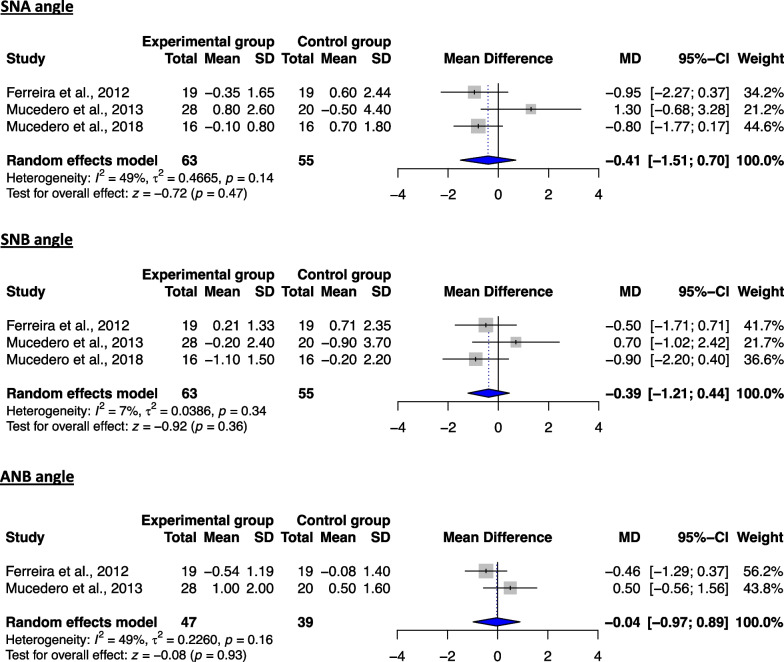
Forest plot of the mean difference of the skeletal sagittal plane angles (in °) between treated and control groups for the T3–T2 interval

Meta-analyses regarding the long-term stability of non-surgical open-bite treatment included data from 3 studies [39, 41, 42] and were performed for 5 skeletal cephalometric variables (3 sagittal and 2 vertical), compared to the untreated group (Table 7). Regarding the vertical plane, no significant differences were found for both respective variables between the two groups (Fig. 2), indicating a relative stability of the corresponding treatment results and prevention of significant relapse. Similar to the latter, no significant differences could be found for any of the evaluated measurements on the sagittal plane (Fig. 3). However, due to the considerable heterogeneity in the majority of the analyses, the respective results should be interpreted with caution. These findings appear to be in agreement with the ones deriving from the qualitative analysis, implying a relative stability of the skeletal changes post-treatment following non-surgical management of the open bite (Table 5).

**Table 7:** Details of the performed meta-analyses for the time intervals T3–T2 and T3–T1

	Variable	k	Effect size			Heterogeneity				
			MD	95% CIs	*P* value	*I*^2^ (%)	*τ*^2^	*Q*	*df*	*P* value
*Time interval: T3–T2*										
1	SNA	3	− 0.41	− 1.51, 0.70	0.47	49.3%	0.47	3.94	2	0.14
2	SNB	3	− 0.39	− 1.21, 0.44	0.36	7.1%	0.04	2.15	2	0.34
3	ANB	2	− 0.04	− 0.97, 0.89	0.93	49%	0.23	1.96	1	0.16
4	NL-ML	2	− 0.12	− 1.22, 0.98	0.83	0	0	0.50	1	0.48
5	ArGoMe	2	− 0.31	− 3.01, 2.39	0.82	79.3%	3.02	4.83	1	0.03
*Time interval: T3–T1*										
1	SNA	4	0.34	− 0.48, 1.16	0.42	54.7	0.38	6.62	3	0.09
2	SNB	4	− 0.03	− 0.60, 0.53	0.90	26	0.09	4.06	3	0.26
3	ANB	4	0.39	− 0.27, 1.05	0.25	46.4	0.21	5.60	3	0.13
4	FH-NL	3	0.44	− 1.66, 2.54	0.68	88.1	3.02	16.82	2	< 0.05
5	NL-ML	4	− 0.67	− 2.58, 1.23	0.49	83.6	3.14	18.31	3	< 0.01
6	FMA	3	0.48	− 1.86, 2.82	0.69	91.3	3.89	22.96	2	< 0.001
7	ArGoMe	3	0.71	− 0.12, 1.54	0.09	4.4	0.03	2.09	2	0.35
8	1s-FH	3	− 1.05	− 3.61, 1.51	0.42	49.4	2.52	3.95	2	0.14
9	1i-ML	3	− 0.84	− 2.62, 0.95	0.36	34.4	0.87	3.05	2	0.22
10	1s-1i	3	2.00	− 2.62, 6.61	0.40	68.2	11.10	6.29	2	0.04
11	**NLA**	**2**	− **3.27**	− **6.51,** − **0.02**	** < 0.05**	**0**	**0**	0.44	1	0.51

The statistical significant changes of the performed meta-analyses are shown in bold

*CI* confidence interval, *Ctr* control, *d.f.* degrees of freedom, *Exp* experimental (treatment), *k* number of included studies in the meta-analysis, *MD* mean difference, *SD* standard deviation

#### Assessment of treatment effects for T3–T1 period (post-retention to the beginning of treatment)

Four studies [37, 38, 41, 42] were possible to be included in at least one meta-analysis regarding this treatment period, including 11 skeletal cephalometric variables (7 skeletal, 3 dentoalveolar and 1 soft tissue) (Table 7).

**Fig. 4:**
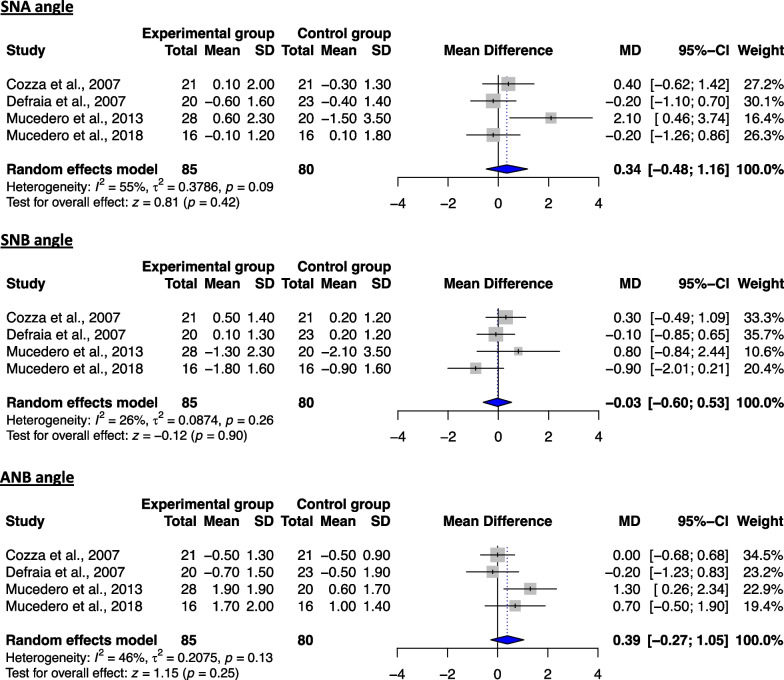
Forest plot of the mean difference of the skeletal sagittal plane angles (in °) between treated and control groups for the T3–T1 interval

**Fig. 5:**
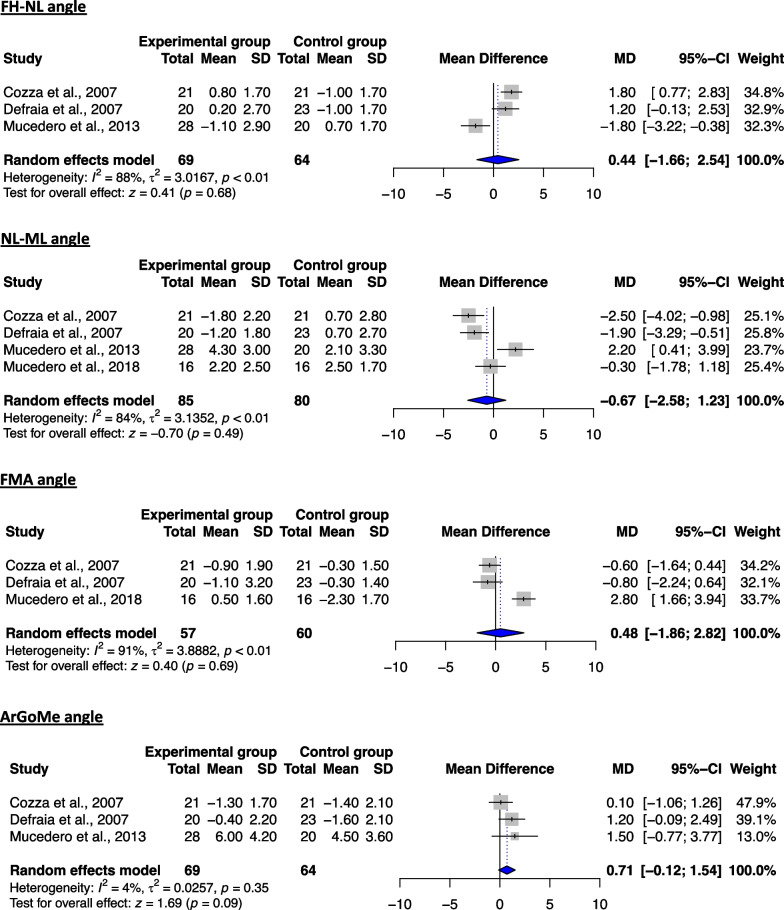
Forest plot of the mean difference of the skeletal vertical plane angles (in °) between treated and control groups for the T3–T1 interval

**Fig. 6:**
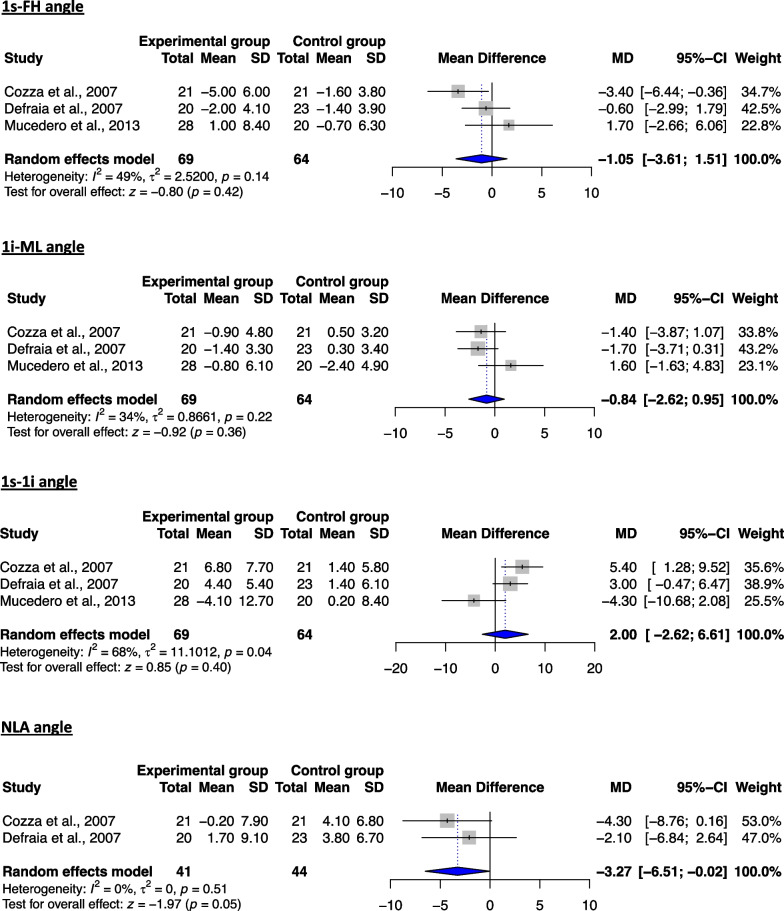
Forest plot of the mean difference of the dentoalveolar and soft tissue angles (in °) between treated and control groups for the T3–T1 interval

As far as the skeletal variables are concerned, no significant differences could be found between the two groups for any of the sagittal or vertical measurements assessed (Figs. 4, 5). This finding rather implies that the assessed treatment modalities do not produce major skeletal changes in the long term and therefore that the correction of the open bite with non-surgical means could be primarily achieved by changes on the dentoalveolar structures on the vertical level. As far as the dentoalveolar measurements are concerned, even though both maxillary and mandibular incisors were found to be more retroclined in the treated patients, the latter failed to reach statistical significance (Fig. 6). In contrast, the nasolabial angle was found to be significantly reduced in the experimental group (Fig. 6). With regard to the qualitative analysis, the results seem to contradict the previously reported outcomes, reporting on favorable changes on the vertical level for the treated patients, yet they should be interpreted with caution (Table 6).

### Risk of bias across studies

Due to inadequate identified studies, an evaluation for the existence of reporting biases was not possible, despite the initial plan. Similarly, the overall quality of the primary outcomes could not be rated according to the GRADE approach.

### Additional analyses

For the same reason, i.e., due to inadequate number of eligible studies, the assessment for possible heterogeneity sources through subgroup analyses or meta-regression was not possible to be conducted. Similarly, sensitivity analyses and an evaluation for the existence of reporting bias (including publication bias) were not performed as well.

## Discussion

### Summary of evidence

This systematic review included data from 244 subjects (134 open-bite patients and 110 untreated individuals), originating from 6 RCTs and non-RCTs, assessing the long-term effectiveness of non-surgical treatment of open bite. Regarding the existing evidence on this subject, there are some pertinent published systematic reviews, which, however, include both controlled and uncontrolled trials in their assessments and respective conclusions [3, 13, 20, 21]. To our knowledge, this is the first meta-analysis including exclusively controlled clinical trials reporting only on post-treatment effects of various open-bite treatment non-surgical alternatives, compared with matched untreated individuals. This inclusion criterion is quite important, since through the comparison of treated versus untreated open-bite patients the pure effects of the therapy (i.e., excluding the effects of normal growth) can be clearly obtained.

In order to assess the exact long-term effects of non-surgical open-bite treatment, the actual normal growth changes produced during the respective active treatment period should be taken into consideration as well. According to the existing literature regarding the treatment effects on anterior open-bite non-surgical treatment, compared to matched untreated open-bite control subjects, from the treatment onset until the removal of the pertinent appliances, the results on the vertical level are rather controversial. In detail, there are studies reporting on no significant skeletal effects [43–46], whereas other studies indicate a significant increase in the palatal plane angle and the angle between the palatal and mandibular planes [41, 47], while finally there are data showing only significant decrease in the mandibular plane angle without important effects on the maxilla [6, 42]. Hence, it could be assumed that the skeletal changes produced by the assessed crib appliances during the active treatment phase on the vertical level are rather minor.

When it comes to the results of the present review, in the long term, skeletal and dentoalveolar effects of non-surgical open-bite treatment, not only are not favorable but rather minimal, both on the vertical and on the sagittal level, suggesting relatively stable results in the long term. The more favorable results for the treated patients were on the soft tissues, as the nasolabial angle had a significant decrease.

More, specifically, concerning the vertical plane and the T3–T2 interval, no significant differences could be found for both assessed variables compared to normal growth, demonstrating a relative stability of the respective treatment effects. This is not in accordance with the findings of Cassis et al. [9], who found a significant decrease in the ArGoMe angle in their control group for the respective treatment interval. However, that particular control group was reported to have a normal occlusion before the onset of the observation period, which is an important confounding factor for the interpretation of the pertinent results. Moreover, a significant relapse of several vertical skeletal measurements after a year of retention, following a three-month magnetic bite-block treatment was reported by Kuster and Ingervall [8], yet again, the respective study did not include an untreated matched control group. In addition, no significant sagittal changes could be identified as well. Unfortunately, no dentoalveolar or soft tissue variables could be included in any statistical analyses for the specific time interval, due to the absence of pertinent data from the included studies.

With regard to the T3–T1 interval, no significant differences could be identified for any of the assessed skeletal measurements. The latter, when taken into account together with the effects produced during the active treatment phase, further reinforces the assumption that the actual skeletal changes that occur during the non-surgical open-bite treatment are not only stable but rather minimal. Thus, it can be assumed that the non-surgical correction of the open bite was accomplished mainly by dentoalveolar changes. However, no significant differences could be identified for any of the sagittal dentoalveolar variables included in the respective meta-analyses, even though the experimental group showed a tendency for more retroclined upper and lower incisors, yet the treatment of non-surgical open bite is long-term effective by means of dentoalveolar correction. Regarding the findings of pertinent existing literature, there are reports that at the end of active open-bite treatment in the mixed dentition, both upper and lower incisors are significantly retroclined and significantly extruded from 1 up to 2 mm per jaw [43, 44, 46, 47]. However, no significant differences were reported in any of the previous studies for the vertical position of both upper and lower molars. Thus, the non-surgical correction of the open bite could be attributed to the exact mode of action of the assessed appliances, i.e., they prevent the mechanical obstacles that are impeding the normal eruption of the incisors [9].

In the present review, no linear variables were considered eligible due to the great probability of magnification bias, and in turn the production of potentially inaccurate conclusions. However, if the magnification factors from the respective radiographic equipment of acquisition could be provided, the corresponding magnification could be taken into account and then the reported linear measurement could be pooled together without the risk of incorrect results.

Moreover, the only significant effect that could be identified in the long term was the reduction in the nasolabial angle for the treated patients, which could be possibly attributed to the retroclination of both upper and lower incisors in the respective patients, even though the latter failed to reach statistical significance. Nevertheless, this finding is not in agreement with two studies [45, 47] reporting on nonsignificant changes between experimental and control groups following active open-bite early treatment.

Another factor that should be pointed out is that in four of the five studies included in the meta-analyses, the examined modalities consisted of crib appliances. The latter is mainly used as an aid to stop deleterious habits such as thumb-sucking or tongue thrust that have been reported as potential etiological factors of anterior open bite [48]. The beneficial effects of these appliances in the conventional management of anterior open bite have been documented in the short-term [13] in matters of significant overbite increase.

Moreover, other innovative treatment alternatives such as temporary anchorage devices (TADs) have been advocated for the non-surgical management of open bite. According to several published studies, the main differences in the treatment philosophy between the latter and the devices assessed in the present review are the fact that TADs are implemented in late adolescent or adults patients with permanent dentition and also that the treatment goal is to intrude the upper molars [49]. The use of skeletal anchorage is reported to successfully correct open bite through maxillary molar intrusion during active treatment [49–51] and only a small relapse is reported at least 2 years post-treatment [50, 51]. However, the respective data originate from uncontrolled studies, implying a need for a cautious interpretation of the pertinent results [50, 51]. Similar to the latter, a pertinent meta-analysis [15] reporting exclusively on long-term treatment results of open-bite correction through skeletal anchorage emphasizes the importance of conducting new controlled trials due to the methodological limitations of the existing relative studies [15].

Hence, when all the latter are taken into account, there is a significant gap of evidence in the literature regarding the effectiveness of open-bite treatment alternatives in the long term and the exact way that the respective treatment outcomes are accomplished. Methodological limitations in pertinent published studies (absence of appropriate control groups, incomplete reporting of cephalometric magnification factors), reporting of specific treatment modalities (i.e., crib appliances) with the combination of a considerable variety of the exact assessed measurements as well as the lack of sufficient relative long-term data prevent the production of robust conclusions and rather lead to assumptions about the exact mode of action of the corresponding devices. Thus, it can only be suspected that open-bite correction through crib appliances seems to be mainly performed through dentoalveolar changes (extrusion) of the anterior teeth through the prevention of the respective deleterious oral habits, yet this assumption has to be appropriately confirmed through further well-designed controlled trials.

### Strengths and limitations

The strengths of this systematic review comprise the predefined protocol, the extensive and unrestricted search of the literature, and the methodology that was strictly and meticulously implemented during every stage of it, according to specific and detailed guidelines [21–23]. In addition, the 5 included studies provided data that enabled well-performed meta-analyses for many important treatment effects. Since a random-effects model was used for data synthesis, the results of the present review provide the average of the open-bite treatment effects across the included studies. Although the majority of studies were conducted in a university setting, the results could be possibly generalized to the average patient, due to the extensive patient inclusion criteria.

Nevertheless, there are also limitations such as the small number of eligible articles that could be identified that permitted the exploration of very few skeletal variables for the T3–T2 interval. For the same reason, the pre-determined additional, sensitivity and quality analyses could not be performed. Moreover, the results of this present review should be interpreted with caution due to the substantial heterogeneity in the majority of the analyses, probably due to variations in patient characteristics and/or the specific treatment protocols followed. In addition, the included studies were found to present methodological limitations that may jeopardize the confidence in the reported outcomes. Moreover, even though the performance of meta-analyses for several variables was feasible, these should be probably considered as exploratory analyses, since they involved data from up to four studies per analysis. Finally, the magnification factors of the cephalometric radiographic devices implemented in the original studies were not reported, which did not permit the inclusion of linear variables in our review, and thus a more detailed evaluation of the produced treatment effects, especially in the vertical level, was not possible.

## Conclusions

According to existing evidence, the following conclusions can be drawn on the effectiveness of non-surgical open-bite treatment in the long term:

1. The skeletal effects of non-surgical open-bite treatment are minimal, both on the vertical and on the sagittal level. Thus, no pertinent differences could be observed for both assessed treatment intervals.
2. Even though both upper and lower incisors were more retroclined compared to the untreated controls, the respective different inclinations failed to reach statistical significance.
3. The only significant long-term change observed following non-surgical open-bite treatment was the reduction in the nasolabial angle in the treated patients.

Taking the latter into account, a need for further well-design studies is quite evident. These should be prospective (ideally randomized) and provide more complete data on patient-related characteristics (gender, skeletal growth stage, and growth pattern of the patients), details of the appliance design, and details of the retention scheme. In addition, beyond the angular measurements, in order to use also linear cephalometric measurements that are prone to magnification bias, the authors should precisely report the magnification factor of the lateral cephalometric radiographs assessed.

## Supplementary Information

**Additional file 1.** Supplementary material.

## Data Availability

The datasets used and/or analyzed during the current study are available from the corresponding author upon reasonable request.
