# Supplementary material for: Long-term effectiveness of non-surgical open-bite treatment: a systematic review and meta-analysis
Source: Prog Orthod. 2023 Jun 1;24:18. doi: 10.1186/s40510-023-00467-2 (PMC10232685; doi:10.1186/s40510-023-00467-2)
Supplement: Supplementary file 1 — Additional file 1. Supplementary material. [file 40510_2023_467_MOESM1_ESM.docx]

**SUPPLEMENTARY MATERIAL**

**Table S1.** The electronic search performed for the identification of eligible studies including the electronic databases explored, the respective search strategies and limitations implemented as well as the corresponding results.

| **Electronic database** | | | **Search strategy used** | | | | **Limits** | **Hits** | | | | |  | | | | | | | |  | | |  | | | | |  |  |  |
| --- | --- | --- | --- | --- | --- | --- | --- | --- | --- | --- | --- | --- | --- | --- | --- | --- | --- | --- | --- | --- | --- | --- | --- | --- | --- | --- | --- | --- | --- | --- | --- |
| **Databases of published trials** | | |  | | | |  |  | | | | |  | | | | | | | |  | | |  | | | | |  |  |  |
| **MEDLINE**  Searched via PubMed on November 20, 2022  http://www.ncbi.nlm.nih.gov/pubmed/advanced | | | (((vertical OR horizontal) AND (axis OR growth OR grower OR pattern OR position OR rotation OR case* OR patient* OR clockwise OR counterclockwise OR (counter-clockwise) OR (counter clockwise) OR component* OR compensat* OR condition* OR condyl* OR correct* OR cranial OR craniofacial OR defect OR deficen* OR deform* OR develop* OR deviat* OR differen* OR dimension* OR direction* OR displacement* OR divergen* OR excess* OR face OR facial OR vector OR posture OR increment* OR (jaw movement) OR (jaw position) OR (jaw relation) OR (lip position) OR deficien* OR measurement* OR measure* OR muscle* OR force* OR (occlusal force*) OR occlusal OR plane OR position* OR profile* OR relation* OR relationship* OR skeletal OR (soft tissue) OR dental OR dentoalveolar OR stability OR stimul* OR tendency OR type)) OR ((open bite) OR (open-bite) OR (openbite*) OR hyperdivergen* OR (long face) OR (long-face) OR (longface*) OR (anterior face height) OR (anterior facial height) OR (posterior face height) OR (posterior facial height))) AND orthodon* AND (stable OR stabil* OR longterm OR (long-term) OR (long term) OR failure* OR fail* OR success* OR retention OR relapse) | | | | *No limitations* | **2501** | | | | |  | | | | | | | |  | | |  | | | | |  |  |  |
| **Scopus**  Searched on November 20, 2022  http://www.scopus.com/search/form.url?display=advanced&clear=t&origin=searchbasic&txGid=1lkb0B3HcbSzUk8cVtIzKL_%3a3 | | | ((open bite) OR (open-bite) OR (openbite*) OR hyperdivergen* OR (long face) OR (long-face) OR (longface*) OR (anterior face height) OR (anterior facial height) OR (posterior face height) OR (posterior facial height)) AND orthodon* AND (stable OR stabil* OR longterm OR (long-term) OR (long term) OR failure* OR fail* OR success* OR retention OR relapse) | | | | *All fields &*  *Limit-to: Sub area"Dent")* | **8436** | | | | |  | | | | | | | |  | | |  | | | | |  |  |  |
| **ScienceDirect**  Searched on November 20, 2022 http://www.sciencedirect.com/science/search | | | All: "open bite" OR "open-bite" OR "openbite" AND  Title, abtract, keywords: stable OR stability OR failure OR success OR retention OR relapse | | | | *Limit to: “Title, Abstract, Keywords”* | **1275** | | | | |  | | | | | | | |  | | |  | | | | |  |  |  |
| **Google Scholar**  Searched on November 20, 2022 https://scholar.google.gr | | | allintitle: stable OR stability OR longterm OR long-term OR failure OR success OR retention OR relapse "open bite"  allintitle: axis OR growth OR grower OR pattern OR position OR rotation OR case OR patient OR clockwise OR counterclockwise OR component OR compensation OR condition OR correction OR cranial OR craniofacial OR defect OR deficiency "open bite"  allintitle: deformation OR development OR deviation OR difference OR direction OR displacement OR divergence OR excess OR measurement OR occlusal OR profile OR relationship OR skeletal OR dental OR stability OR tendency OR type "open bite" | **209** | | | *Limit to: “Title”* | **1725** | | | | |  | | | | |  | | | | | |  | | | | |  |  |  |
|  |  |  |  | **770** | | |  |  |  |  |  |  |  |  |  |  |  |  |  |  |  |  |  |  |  |  |  |  |  |  |  |
|  |  |  |  | **746** | | |  |  |  |  |  |  |  |  |  |  |  |  |  |  |  |  |  |  |  |  |  |  |  |  |  |
| **Web of Science**  Searched on November 20, 2022  http://apps.webofknowledge.com/WOS_AdvancedSearch_input.do?SID=C5rSx1xxt6rarqFA6vb&product=WOS&search_mode=AdvancedSearch | | | (TS=((open bite) OR (open-bite) OR (openbite*) OR hyperdivergen* OR (long face) OR (long-face) OR (longface*) OR (anterior face height) OR (anterior facial height) OR (posterior face height) OR (posterior facial height)) AND TS=(stable OR stabil* OR longterm OR (long-term) OR (long term) OR failure* OR fail* OR success* OR retention* OR relapse*)) OR (TS=(vertical OR horizontal) AND TS= (axis OR growth OR pattern OR patient* OR component* OR defect* OR deficen* OR deform* OR develop*OR direction* OR excess* OR position* OR profile* OR relation* OR tenden* OR type*)) AND TS=(orthodon*) | | | | *No limitations* | **2748** | | | | |  | | | | |  | | | | | |  | | | | |  |  |  |
| **Cochrane Database of Systematic Reviews**  Searched via The Cochrane Library  on November 20, 2022  https://www.cochranelibrary.com/advanced-search | | | ((open bite) OR (open-bite) OR (openbite*) OR hyperdivergen* OR (long face) OR (long-face) OR (longface*)) AND (stable OR stabil* OR failure* OR fail* OR retention OR relapse) | | | | *No limitations* | **1739** | | | | |  | | | | | | | |  | | |  | | | | |  |  |  |
| **Cochrane Central Register of Controlled Trials**  Searched via The Cochrane Library  on November 20, 2022 https://www.cochranelibrary.com/advanced-search | | | ((open bite) OR (open-bite) OR (openbite*) OR hyperdivergen* OR (long face) OR (long-face) OR (longface*)) AND (stable OR stabil* OR failure* OR fail* OR retention OR relapse) | | | | *No limitations* | **714** | | | | |  | | | | | | | |  | | |  | | | | |  |  |  |
| **Ovid database**  Searched via HEAL-Link on November 20, 2022  http://ovidsp.ovid.com | | | (((vertical OR horizontal) AND (axis OR growth OR grower OR pattern OR position OR rotation OR case* OR patient* OR clockwise OR counterclockwise OR (counter-clockwise) OR (counter clockwise) OR component* OR compensat* OR condition* OR condyl* OR correct* OR cranial OR craniofacial OR defect OR deficen* OR deform* OR develop* OR deviat* OR differen* OR dimension* OR direction* OR displacement* OR divergen* OR excess* OR face OR facial OR vector OR posture OR increment* OR (jaw movement) OR (jaw position) OR (jaw relation) OR (lip position) OR deficien* OR measurement* OR measure* OR muscle* OR force* OR (occlusal force*) OR occlusal OR plane OR position* OR profile* OR relation* OR relationship* OR skeletal OR (soft tissue) OR dental OR dentoalveolar OR stability OR stimul* OR tendency OR type)) OR ((open bite) OR (open-bite) OR (openbite*) OR hyperdivergen* OR (long face) OR (long-face) OR (longface*) OR (anterior face height) OR (anterior facial height) OR (posterior face height) OR (posterior facial height))) AND orthodon* AND (stable OR stabil* OR longterm OR (long-term) OR (long term) OR failure* OR fail* OR success* OR retention OR relapse) | | | | *Limit to: «Title, abstract, keywords»* | **2743** | | | | |  | | | | | | | |  | | |  | | | | |  |  |  |
| **VHL Search Portal**  Searched on November 20, 2022  (Databases included: LILACS, BBO- Dentistry, IBECS, BINACIS, MedCarib)  http://pesquisa.bvsalud.org/portal/advanced/?lang=en | | | (((vertical OR horizontal) AND (axis OR growth OR grower OR pattern OR position OR rotation OR case* OR patient* OR clockwise OR counterclockwise OR (counter-clockwise) OR (counter clockwise) OR component* OR compensat* OR condition* OR condyl* OR correct* OR cranial OR craniofacial OR defect OR deficen* OR deform* OR develop* OR deviat* OR differen* OR dimension* OR direction* OR displacement* OR divergen* OR excess* OR face OR facial OR vector OR posture OR increment* OR (jaw movement) OR (jaw position) OR (jaw relation) OR (lip position) OR deficien* OR measurement* OR measure* OR muscle* OR force* OR (occlusal force*) OR occlusal OR plane OR position* OR profile* OR relation* OR relationship* OR skeletal OR (soft tissue) OR dental OR dentoalveolar OR stability OR stimul* OR tendency OR type)) OR ((open bite) OR (open-bite) OR (openbite*) OR hyperdivergen* OR (long face) OR (long-face) OR (longface*) OR (anterior face height) OR (anterior facial height) OR (posterior face height) OR (posterior facial height))) AND orthodon* AND (stable OR stabil* OR longterm OR (long-term) OR (long term) OR failure* OR fail* OR success* OR retention OR relapse) | | | | *Limit to: “Title, abstract, subject”* | **258** | | | | |  | | | | | | | |  | | |  | | | | |  |  |  |
| **Evidence-Based Medicine**  Searched on November 20, 2022 http://ebm.bmj.com/search | | | Abstract or Title: ((open bite) OR (open-bite) OR (openbite)) AND  Full Text or Abstract or Title: (stable OR stabil* OR failure* OR fail* OR success* OR retention OR relapse**)** | | | | *(select “any”)* | **1664** | | | | | | | | | | | | | | | | | | | | | | | |
| **Nature Databases and Gateways**  Searched on November 20, 2022  http://www.nature.com/search/advanced?sp-a=sp1001702d&sp-x-1=ujournal | | | (((vertical OR horizontal) AND (axis OR growth OR grower OR pattern OR position OR rotation OR case* OR patient* OR clockwise OR counterclockwise OR (counter-clockwise) OR (counter clockwise) OR component* OR compensat* OR condition* OR condyl* OR correct* OR cranial OR craniofacial OR defect OR deficen* OR deform* OR develop* OR deviat* OR differen* OR dimension* OR direction* OR displacement* OR divergen* OR excess* OR face OR facial OR vector OR posture OR increment* OR (jaw movement) OR (jaw position) OR (jaw relation) OR (lip position) OR deficien* OR measurement* OR measure* OR muscle* OR force* OR (occlusal force*) OR occlusal OR plane OR position* OR profile* OR relation* OR relationship* OR skeletal OR (soft tissue) OR dental OR dentoalveolar OR stability OR stimul* OR tendency OR type)) OR ((open bite) OR (open-bite) OR (openbite*) OR hyperdivergen* OR (long face) OR (long-face) OR (longface*) OR (anterior face height) OR (anterior facial height) OR (posterior face height) OR (posterior facial height))) AND orthodon* AND (stable OR stabil* OR longterm OR (long-term) OR (long term) OR failure* OR fail* OR success* OR retention OR relapse) | | | | No limitations | **951** | | | | |  | | | | |  | | | | | |  | | | | |  |  |  |
| **African Journals Online**  Searched on November 20, 2022  http://www.ajol.info/index.php/index/search | | | (open bite) OR (open-bite) OR (openbite*) OR hyperdivergen* OR (long face) OR (long-face) OR (longface*) OR (anterior face height) OR (anterior facial height) OR (posterior face height) OR (posterior facial height) | | | | *No limitations* | **18** | | | | |  | | | | |  | | | | | |  | | | | |  |  |  |
| **Databases of dissertations, theses and conference proceedings** | | | | | | | | | | |  | | | | |  | | | | | |  | | | | |  |  |  |  |  |
| **ProQuest**  Searched on November 20, 2022  http://proquest.umi.com/pqdweb?RQT=403& TS=1321887206&clientId=68919 | | (((vertical OR horizontal) AND (axis OR growth OR grower OR pattern OR position OR rotation OR case* OR patient* OR clockwise OR counterclockwise OR (counter-clockwise) OR (counter clockwise) OR component* OR compensat* OR condition* OR condyl* OR correct* OR cranial OR craniofacial OR defect OR deficen* OR deform* OR develop* OR deviat* OR differen* OR dimension* OR direction* OR displacement* OR divergen* OR excess* OR face OR facial OR vector OR posture OR increment* OR (jaw movement) OR (jaw position) OR (jaw relation) OR (lip position) OR deficien* OR measurement* OR measure* OR muscle* OR force* OR (occlusal force*) OR occlusal OR plane OR position* OR profile* OR relation* OR relationship* OR skeletal OR (soft tissue) OR dental OR dentoalveolar OR stability OR stimul* OR tendency OR type)) OR ((open bite) OR (open-bite) OR (openbite*) OR hyperdivergen* OR (long face) OR (long-face) OR (longface*) OR (anterior face height) OR (anterior facial height) OR (posterior face height) OR (posterior facial height))) AND orthodon* AND (stable OR stabil* OR longterm OR (long-term) OR (long term) OR failure* OR fail* OR success* OR retention OR relapse) | | | | *Limit to: “Anywhere except full text”* | | | | **656** | | | | |  | | | | |  | | | | | |  | | | | |  |
| **German National Library of Medicine (ZB MED)**  Searched via MEDPILOT on November 20, 2022 https://www.livivo.de/app/search/filter?field=DB&mode=add&value=KOELN | | keyword: ((open bite) OR (open-bite) OR (openbite*) OR hypodivergen* OR (long face) OR (long-face) OR (longface*) OR (facial height)) AND (stable OR stabil* OR failure* OR fail* OR retention* OR relapse) | | | | *Limit to: “Catalogue medicine. health”* | | | | **707** | | | | |  | | | | |  | | | | | |  | | | | |  |
| **Databases of research registers** | | | | | | | | | | | |  | | | | |  | | | | | |  | | | | |  |  |  |  |
| ***meta*Register of Controlled Trials**  Searched on November 20, 2022 http://www.isrctn.com/editAdvancedSearch | | (open bite) OR (open-bite) OR (openbite*) OR hyperdivergen* | | | | *Limit to:* “Text search” | | | | **17** | | | | |  | | | | |  | | | | | |  | | | | |  |
| **ClinicalTrials.gov**  Searched on November 20, 2022 https://clinicaltrials.gov/ | | (open bite) OR (open-bite) OR (openbite*) OR hyperdivergen* | | | | *Limit to:* “Condition or Disease” | | | | **92** | | | | |  | | | | |  | | | | | |  | | | | |  |
| **Sum** |  | | | |  | | | | **26244** | | | | |  | | | | |  | | | | | |  | | | | |  |  |

**Table S2.** Classification of the angular cephalometric variables examined in the present study, with the different terms used among the selected studies, and their definitions.

| **Term used** | **Other terms** | **Definition** |
| --- | --- | --- |
| ***Skeletal variables*** | | |
| SNA | - | The posterior-inferior angle formed by Se, Na and A point |
| SNB | - | The inferior-posterior angle defined by Se, Na and B point |
| ANB | - | The angle between Point A, Na and Point B; the difference between angles SNA and SNB |
| SN.GoGn | - | The angle formed by the anterior cranial base plane and the mandibular plane |
| FH-NL | FH-PP | The angle formed between the Frankfort horizontal and the Palatal plane |
| SN.PP | SN-PP | The angle between the Sella-Nasion line and the Palatal plane |
| NL-ML | Palatal plane to mandibular plane, PP-MP | The angle formed by the Palatal plane and the Mandibular plane |
| ArGoMe | Gonial angle, Go | The angle formed by Ar, Go and Me |
| FMA | MPA | The anterior-inferior angle formed by the Frankfort Horizontal line and the Mandibular plane |
| NS.Gn | - | The angle formed by the SN plane and the SGn plane indicating the vertical and anteroposterior mandibular growth |
| The total of the facial angles (Σ) (Jarabak) | - | The polygon formed by the Nasion, sella, articulare, gonion, and gnathion points |
| ***Dentoalveolar variables*** | | |
| 1s-FH | U1-FH | Angle between the axis of the upper incisor and FH line |
| U1.NA | - | Angle between the upper incisor long axis and the NA line |
| 1s-1i | Interincisal angle | The angle formed by the long axis of the upper and lower incisors |
| 1i-ML | L1-MPA | Angle between lower incisor long axis and mandibular plane |
| L1.NB | - | Angle between lower incisor long axis and the NB line |
| ***Soft tissue variables*** | | |
| NLA | Nasolabial angle | Angle between the nasal line and the most anterior point of the upper lip |
| Mentolabial angle | - | Angle between the line of the lower lip and the Me' |
| Gl.Sn.P’ | - | The angle formed by Ga', Sn and Pg' indicating the facial convexity |

**Table S3.** Number of excluded studies with reasons.

| **Reason for exclusion** | **Excluded articles on the basis of title and abstract** | **Excluded articles on the basis of full text** |
| --- | --- | --- |
| Irrelevant to the subject of the review | 12942 | 131 |
| Animal studies | 25 | - |
| Patients with syndromes or CLP | 147 | - |
| Patients with TMD | 84 | - |
| Patients with deep bite | 39 | - |
| Studies without long term results on open bite | 12 | 64 |
| Studies providing only linear measurements on lateral cephalograms | 2 | 8 |
| Studies with missing or inappropriate data | 5 | 99 |
| Electromyographic evaluation | 20 | - |
| Unsupported opinion of expert | 7 | 3 |
| Replies to the author/editor | 13 | 4 |
| Interviews | 1 | - |
| Commentaries | 14 | 1 |
| Books’/conferences’ abstracts | 200 | 3 |
| Summaries | 49 | 1 |
| In vitro/In silico studies | 4 | - |
| Cross-sectional surveys | 3 | - |
| Case series without a control or with inappropriate control group | 665 | 63 |
| Case reports or reports of cases | 18 | 9 |
| Case–control observational studies | 3 | - |
| Cohort studies | 7 | - |
| Narrative reviews | 56 | 26 |
| Systematic reviews | 61 | 1 |
| Meta-analyses | 21 | - |
| No English abstract | 12 | - |
| Surgical treatment | 87 | - |
| **Sum** | **14497** | **413** |

## Table S4a. Risk of bias assessment checklist for randomised studies (Rob 2.0)

| Signalling questions | Ferreira et al. [39] |
| --- | --- |
| **Domain 1: Risk of bias arising from the randomization process** | |
| 1.1 Was the allocation sequence random? | NI |
| 1.2 Was the allocation sequence concealed until participants were enrolled and assigned to interventions? | NI |
| 1.3 Did baseline differences between intervention groups suggest a problem with the randomization process? | N |
| Risk-of-bias judgement | Some concerns |
| **Domain 2: Risk of bias due to deviations from the intended interventions (effect of assignment to intervention)** | |
| 2.1. Were participants aware of their assigned intervention during the trial? | NI |
| 2.2. Were carers and people delivering the interventions aware of participants' assigned intervention during the trial? | NI |
| 2.3. If Y/PY/NI to 2.1 or 2.2: Were there deviations from the intended intervention that arose because of the trial context? | NI |
| 2.4 If Y/PY to 2.3: Were these deviations likely to have affected the outcome? | NA |
| 2.5. If Y/PY/NI to 2.4: Were these deviations from intended intervention balanced between groups? | NA |
| 2.6 Was an appropriate analysis used to estimate the effect of assignment to intervention? | PN |
| 2.7 If N/PN/NI to 2.6: Was there potential for a substantial impact (on the result) of the failure to analyse participants in the group to which they were randomized? | N |
| Risk-of-bias judgement | Some Concerns |
| **Domain 3: Missing outcome data** | |
| 3.1 Were data for this outcome available for all, or nearly all, participants randomized? | PY |
| 3.2 If N/PN/NI to 3.1: Is there evidence that the result was not biased by missing outcome data? | NA |
| 3.3 If N/PN to 3.2: Could missingness in the outcome depend on its true value? | NA |
| 3.4 If Y/PY/NI to 3.3: Is it likely that missingness in the outcome depended on its true value? | NA |
| Risk-of-bias judgement | Low |
| **Domain 4: Risk of bias in measurement of the outcome** | |
| 4.1 Was the method of measuring the outcome inappropriate? | Y |
| 4.2 Could measurement or ascertainment of the outcome have differed between intervention groups? | N |
| 4.3 If N/PN/NI to 4.1 and 4.2: Were outcome assessors aware of the intervention received by study participants? | NA |
| 4.4 If Y/PY/NI to 4.3: Could assessment of the outcome have been influenced by knowledge of intervention received? | NA |
| 4.5 If Y/PY/NI to 4.4: Is it likely that assessment of the outcome was influenced by knowledge of intervention received? | NA |
| Risk-of-bias judgement | Low |
| **Domain 5: Risk of bias in selection of the reported result** | |
| 5.1 Were the data that produced this result analysed in accordance with a pre-specified analysis plan that was finalized before unblinded outcome data were available for analysis? | NI |
| Is the numerical result being assessed likely to have been selected, on the basis of the results, from... |  |
| 5.2. ... multiple eligible outcome measurements (e.g. scales, definitions, time points) within the outcome domain? | PN |
| 5.3 ... multiple eligible analyses of the data? | PN |
| Risk-of-bias judgement | Some concerns |
| **Overall risk of bias** | |
| Risk-of-bias judgement | High |

*Responses Y/PY are potential markers for low risk of bias, and responses PN/N are potential markers for a risk of bias.

## Table S4b. Risk of bias assessment checklist for non-randomized trials (ROBINS-I)

| Signalling questions | Cozza et al. [37] | Defraia et al. [38] | Frankel and Frankel [40] | Mucedero et al. [41] | Mucedero et al. [42] |
| --- | --- | --- | --- | --- | --- |
| **1. Bias due to confounding** | | | | | |
| 1.1 Is there potential for confounding of the effect of intervention in this study? | PN | PN | PY | PN | PN |
| 1.2. Was the analysis based on splitting participants’ follow up time according to intervention received? | NA | NA | PN | NA | NA |
| 1.3. Were intervention discontinuations or switches likely to be related to factors that are prognostic for the outcome? | NA | NA | NA | NA | NA |
| ***Questions relating to baseline confounding only*** |  |  |  |  |  |
| 1.4. Did the authors use an appropriate analysis method that controlled for all the important confounding domains? | NA | NA | NI | NA | NA |
| 1.5. **If Y/PY to 1.4**: Were confounding domains that were controlled for measured validly and reliably by the variables available in this study? | NA | NA | NA | NA | NA |
| 1.6. Did the authors control for any post-intervention variables that could have been affected by the intervention? | NA | NA | NI | NA | NA |
| ***Questions relating to baseline and time-varying confounding*** |  |  |  |  |  |
| 1.7. Did the authors use an appropriate analysis method that controlled for all the important confounding domains and for time-varying confounding? | NA | NA | NI | NA | NA |
| 1.8. **If Y/PY to 1.7**: Were confounding domains that were controlled for measured validly and reliably by the variables available in this study? | NA | NA | NA | NA | NA |
| ***Risk of bias judgement*** | Low | Low | Moderate risk | Low | Low |
| **2. Bias in selection of participants into the study** | | | | | |
| 2.1. Was selection of participants into the study (or into the analysis) based on participant characteristics observed after the start of intervention? **If N/PN to 2.1:** go to 2.4 | N | N | N | N | N |
| 2.2. **If Y/PY to 2.1**: Were the post-intervention variables that influenced selection likely to be associated with intervention? | NA | NA | NA | NA | NA |
| 2.3 **If Y/PY to 2.2**: Were the post-intervention variables that influenced selection likely to be influenced by the outcome or a cause of the outcome? | NA | NA | NA | NA | NA |
| 2.4. Do start of follow-up and start of intervention coincide for most participants? | NI | NI | NI | NI | NI |
| 2.5. **If Y/PY to 2.2 and 2.3, or N/PN to 2.4**: Were adjustment techniques used that are likely to correct for the presence of selection biases? | NA | NA | NA | NA | NA |
| ***Risk of bias judgement*** | Low | Low | Low | Low | Low |
| **3. Bias in classification of interventions** | | | | | |
| 3.1 Were intervention groups clearly defined? | Y | Y | Y | Y | Y |
| 3.2 Was the information used to define intervention groups recorded at the start of the intervention? | Y | Y | Y | Y | Y |
| 3.3 Could classification of intervention status have been affected by knowledge of the outcome or risk of the outcome? | N | N | N | N | N |
| ***Risk of bias judgement*** | Low | Low | Low | Low | Low |
| **4. Bias due to deviations from intended interventions** | | | | | |
| 4.1. Were there deviations from the intended intervention beyond what would be expected in usual practice? | PN | PN | PN | PN | PN |
| 4.2. **If Y/PY to 4.1**: Were these deviations from intended intervention unbalanced between groups *and* likely to have affected the outcome? | NA | NA | NA | NA | NA |
| ***Risk of bias judgement*** | Low | Low | Low | Low | Low |
| **5. Bias due to missing data** | | | | | |
| 5.1 Were outcome data available for all, or nearly all, participants? | PY | PY | PY | PY | PY |
| 5.2 Were participants excluded due to missing data on intervention status? | PN | PN | PN | PN | PN |
| 5.3 Were participants excluded due to missing data on other variables needed for the analysis? | PN | PN | PN | PN | PN |
| 5.4 **If PN/N to 5.1, or Y/PY to 5.2 or 5.3**: Are the proportion of participants and reasons for missing data similar across interventions? | NA | NA | NA | NA | NA |
| 5.5 **If PN/N to 5.1, or Y/PY to 5.2 or 5.3**: Is there evidence that results were robust to the presence of missing data? | NA | NA | NA | NA | NA |
| ***Risk of bias judgement*** | Low | Low | Low | Low | Low |
| **6. Bias in measurement of outcomes** | | | | | |
| 6.1 Could the outcome measure have been influenced by knowledge of the intervention received? | N | N | N | N | N |
| 6.2 Were outcome assessors aware of the intervention received by study participants? | NI | NI | NI | NI | NI |
| 6.3 Were the methods of outcome assessment comparable across intervention groups? | PY | PY | PY | PY | PY |
| 6.4 Were any systematic errors in measurement of the outcome related to intervention received? | PN | PN | PN | PN | PN |
| ***Risk of bias judgement*** | Low | Low | Low | Low | Low |
| **7. Bias in selection of the reported result** | | | | | |
| Is the reported effect estimate likely to be selected, on the basis of the results, from... |  |  |  |  |  |
| 7.1. ... multiple outcome *measurements* within the outcome domain? | PN | PN | PN | PN | PN |
| 7.2 ... multiple *analyses* of the intervention-outcome relationship? | PN | PN | PN | PN | PN |
| 7.3 ... different *subgroups*? | PN | PN | PN | PN | PN |
| ***Risk of bias judgement*** | Low | Low | Low | Low | Low |
| **Overall bias** | | | | | |
| ***Risk of bias judgement*** | Low | Low | Moderate risk | Low | Low |

*Responses Y/PY are potential markers for low risk of bias, and responses PN/N are potential markers for a risk of bias.
